# Non-Invasive Detection of Anti-Inflammatory Bioactivity and Key Chemical Indicators of the Commercial Lanqin Oral Solution by Near Infrared Spectroscopy

**DOI:** 10.3390/molecules27092955

**Published:** 2022-05-05

**Authors:** Hui Ma, Lulu Xiao, Dongchen Xu, Yingrui Geng, Xuesong Liu, Yong Chen, Yongjiang Wu

**Affiliations:** College of Pharmaceutical Sciences, Zhejiang University, Hangzhou 310058, China; 12119013@zju.edu.cn (H.M.); 22119162@zju.edu.cn (L.X.); xdcgg@zju.edu.cn (D.X.); 22019084@zju.edu.cn (Y.G.); liuxuesong@zju.edu.cn (X.L.)

**Keywords:** Chinese medicine formulations, non-invasive detection, near infrared spectroscopy, Lanqin oral solution, anti-inflammatory, epigoitrin, geniposide, baicalin

## Abstract

Quality control methods of current traditional Chinese medicine (TCM) preparation is time-consuming and difficult to assess in terms of overall efficiency of the drug. A non-destructive rapid near-infrared spectroscopy detection system for key chemical components and biological activity of Lanqin oral solution (LOS), one of the best-selling TCM formulations, was established for comprehensive quality evaluation. Near infrared spectral scanning was carried out on 101 batches of commercial LOS under the penetrated vial state and traditional state. RAW 264.7 cells were cultured to detect the anti-inflammatory ability of LOS, and the reference concentrations of epigoitrin, geniposide, and baicalin were obtained by HPLC. The quantitative models were optimized by three kinds of variable selection methods. The correlation coefficients of prediction value of the models were greater than 0.94. The system also passed the external validation. The performance of the non-invasive models was similar to the traditional models. The established non-destructive system can be applied to the rapid quality inspection of LOS to avoid unqualified drugs from entering the market and ensure drug effectiveness. The biological activity index of LOS was introduced and predicted by NIRs for the first time, which provides a new idea about the quality control of TCM formulations.

## 1. Introduction

Enterprises and government departments have always been concerned with the quality consistency control of drugs. For finished pharmaceutical formulations, the current testing methods need to damage the container, which is destructive to the product [1]. As a result, it is impossible for the tested samples to enter the subsequent commercial circulation. Therefore, the finished products can only be analyzed by sampling [2]. At the same time, the traditional analytical method has the limitations of human and material resource consumption and is time-consuming, which reduces the production efficiency of enterprises and deviates from the demand for efficient and continuous production [3]. To prevent substandard drugs from entering the market and causing delays in the production cycle of manufacturers, it is necessary to develop a non-destructive, vial-penetrating, and rapid detection method.

Near-infrared spectroscopy (NIRs) has been widely applied in the food, petroleum, and pharmaceutical fields due to its advantages of rapidity, non-destructiveness, and lack of a need for sample pretreatment [4,5,6]. The information of multiple indicators in the sample system can be parsed by NIRs, which meets the requirements of multiple efficacy indicators of traditional Chinese medicine (TCM) [7,8]. Combined with chemometric methods, the feasibility of NIRs in detecting TCM formulations has been demonstrated. Yan et al., created a rapid quality-assessment system for Chinese medicine preparation Honghua Oil [9]. Si et al., achieved qualitative and quantitative analysis of Yaobitong capsule without damaging capsule shell [10]. However, NIRs are mostly applied for rapid detection of chemical indicators of drugs, and its application potential in the detection of drug biological activity remains to be tapped.

Compared with chemical formulations, components of TCM formulations are complex. TCM formulations are usually prepared by decoction of herbs, and the system contains hundreds of components. At the same time, the mechanism of TCM has yet to be explored [11]. It is difficult to reflect the overall efficiency of the drug by analyzing the concentration of specific components [12]. Lanqin oral solution (LOS) is famous for its heat-clearing and detoxifying properties, and is commonly used in the treatment of pharyngitis. Clinical experiments have shown that taking LOS has a significant therapeutic effect on children with herpangina, and it can shorten the fade time of fever and herpes without increasing the occurrence of adverse reactions [13]. There is research finding that LOS can shorten the healing time of acute pharyngitis [14]. For patients with chronic pharyngitis, Li et al., conducted a randomized controlled trial on 1642 patients. The results of the meta-analysis revealed that LOS can effectively inhibit the increase of various inflammatory factors and is beneficial to the relief of patients’ symptoms [15]. However, the therapeutic efficiency mechanism of LOS has not yet been elucidated. LOS is made of *Isatidis Radix*, *Gardeniae Fructus*, *Scutellariae Radix*, *Phellodendri Chinensis Cortex*, and *Sterculiae Lychnophorae Semen.* The quality-control method research of LOS mainly focuses on three substances: epigoitrin, geniposide, and baicalin [16]. The efficiency of TCM is considered to be the result of the synergistic effect of multiple components [17]. It is incomplete to assess the quality by focusing on single or several chemical substances in TCM as single substance is inadequate to ensure the efficiency of LOS. The material basis of LOS is complex. Studies have found that there are at least 175 chemical components in the LOS system [18]. It is impractical to separate and analyze all substances in LOS. Therefore, it is worthwhile to evaluate the quality of LOS directly through pharmacodynamic indicators.

The aim of this study was to achieve a comprehensive quality assessment of LOS without destroying the vial. In this study, an inflammatory model was constructed by lipopolysaccharides (LPS)-stimulated cells to investigate the anti-inflammatory ability of LOS. At the same time, HPLC analysis was performed on 101 batches of drugs to analyze the concentration of epigoitrin, geniposide, and baicalin in the sample. The NIR spectra of the LOS collected with the container and under the traditional state were collected to construct the optimal partial least squares regression (PLSR) models. It was expected that this research was the first attempt at a non-invasive rapid detection system for drug activity of LOS.

## 2. Results

### 2.1. Raw Spectra Analysis

**Figure 1:**
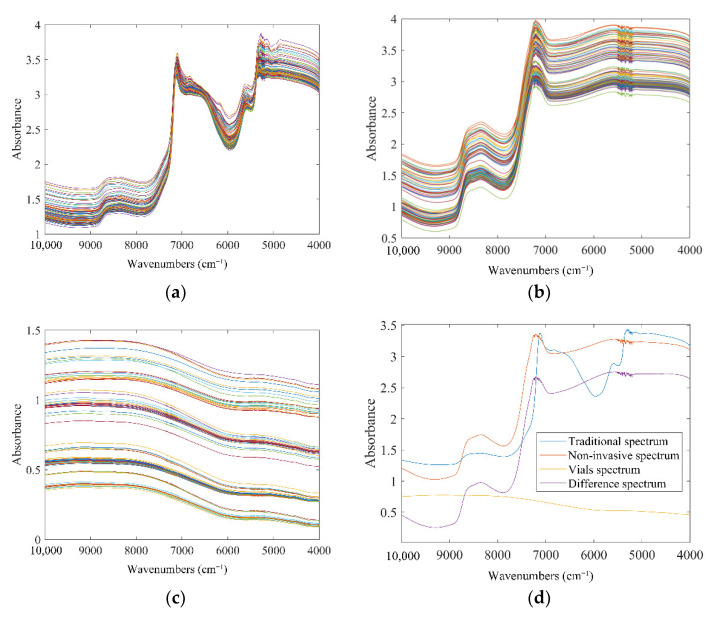
The raw NIR absorbance spectra: (**a**) traditional spectra; (**b**) spectra that penetrates the vial; (**c**) spectra of the vial; (**d**) average spectra.

The raw NIR spectra of 101 samples in scanning tubes or commercial vials are shown in Figure 1a,b. It was apparent that the raw spectra of samples collected from different production batches were overall similar in both states. The obvious bands around 7000 cm^−1^ were generated by the first overtone of the O-H stretching vibration of water. Additionally, its combined absorption band with the second overtone can be observed near 5100 cm^−1^. The broad band extending from 8800 cm^−1^ to 8000 cm^−1^ was consistent with the second overtone region of bonded C-H. The NIR spectra of the empty vials are displayed in Figure 1c. The main components of the glass bottles were silica and other inorganic substances, so their NIR spectra were gentle lines without obvious absorption bands. The spectra of 101 vials fluctuated due to certain differences in quality between vials. It corresponded to the realistic scenario of spectral applications. The purple line in Figure 1d is the difference spectrum, calculated by subtracting the spectrum of the corresponding empty vial from the sample spectrum penetrated the vial. Compared with the red line in the figure, there was no significant difference between the two. It suggested the feasibility of non-invasive models. The directly observable bands in Figure 1 were caused by C, H, O elements that were widely present in various substances. It was difficult to directly correlate spectral features with the concerns. Therefore, it was necessary to introduce a chemometric method to further mine the information in the spectra to realize the non-destructive detection of LOS.

### 2.2. Reference Data Analysis

**Figure 2:**
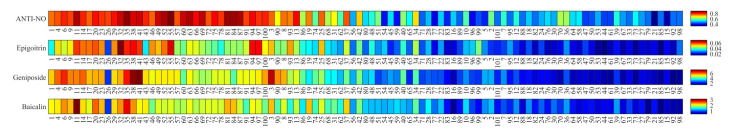
Heat map of 4 chosen targets.

The production years of the collected samples spanned 3 years, from July 2019 to July 2021. The anti-inflammatory ability and content distribution of key chemical indicators of 101 batches of LOS are displayed in Figure 2. The color of the sample gradually changed from red to blue as the value decreases, which was shown on the right side of the figure. Sample No. 100 and the samples on the left side were 35 batches of samples without pre-dilution, and the samples to the right of No. 100 were sorted to the right by increasing dilution. It can be observed that the reference value of the sample decreased roughly with the increase of the dilution factor. The distance correlation analysis was performed between the inhibition rate of nitric oxide production (ANTI-NO) and key chemical indicators of the 101 samples, and the *p* values of epigoitrin, geniposide, and baicalin were 0.8610, 0.901, and 0.912, respectively. The three chemical indicators selected based on the experience had significant correlations with the biological activity of the sample. It was proved that the selection of chemical detection indicators was reasonable. From the distribution of the reference values of the first 35 batches of samples, it can be found that even without human intervention, the quality of the LOS still fluctuated from batch to batch. The concentration of epigoitrin, geniposide, and baicalin in these 35 batches ranged from 0.02546–0.0702 mg/mL, 2.388–7.413 mg/mL, and 0.9230–3.131 mg/mL, respectively. In contrast, ANTI-NO fluctuated steadily in the range of 75.55% to 88.97%. The concentration of epigoitrin, geniposide, and baicalin fluctuated within a 3-fold range, while the corresponding biological activity was stable in the range of 15%, which revealed that the current detection method based only on chemical indicators was not enough to achieve the comprehensive quality control of TCM formulations. It was necessary to further introduce the detection of pharmacodynamics indicators on the basis of the existing detection methods. However, the detection of anti-inflammatory activity relied on cells and will take at least 3 days. Traditional detection methods will greatly increase the burden on enterprises. Therefore, it was necessary to establish a system that can complete the rapid detection of biological activity and key chemical components simultaneously.

### 2.3. Model Construction under Traditional State

#### 2.3.1. Sample Sets Division

Each dataset was divided into a calibration set with 67 samples and a prediction set with 23 samples by the SPXY algorithm. The reference value ranges for each dataset are listed in Table 1. It was worth noting that the mean value of the prediction sets was close to the calibration set for each dataset, which proved that the properties of the two datasets were similar and the data division was reasonable. At the same time, the ranges of target indexes in the prediction sets were covered within that of the calibration sets, which was beneficial to NIR models. Sample numbers 3, 6, 22, 32, 34, 35, 40, 55, 91, 92, 99 were randomly selected as the external validation set. Among them, 5 were LOS original samples and 6 were artificially diluted samples. It can be observed that the fluctuation range of the validation set of ANTI-NO, epigoitrin, and geniposide all exceeded the calibration set, which was a challenge to the established PLSR model.

#### 2.3.2. Spectral Pretreatment and Variables Selection

The performances of different spectral pretreatments models for each indicator are recorded in Table S2. The optimal pretreatment was chosen according to the model parameters of the calibration set. The normalization pretreated spectra obtained high correlation coefficients of calibration (Rc)values and low relative standard error of calibration (RSEC) values for ANTI-NO and baicalin. For epigoitrin and geniposide, meanwhile, the optimal pretreatment was SG smoothing and MSC, respectively. After preprocessing, the baseline drift between different samples had been compressed. The Rc values of the prediction models of ANTI-NO, epigoitrin, geniposide, and baicalin increased from 0.9305, 0.8989, 0.9802, 0.9265 to 0.9491, 0.9119, 0.9803, 0.9203, respectively. The relative standard error of prediction (RSEP) values representing the model prediction errors decreased from 10.6%, 15.1%, 8.8%, and 19.9% to 9.8%, 14.9%, 8.6%, and 18.5%, respectively. The improvements of the performances of the models demonstrated that the noise in the system had been removed.

**Figure 3:**
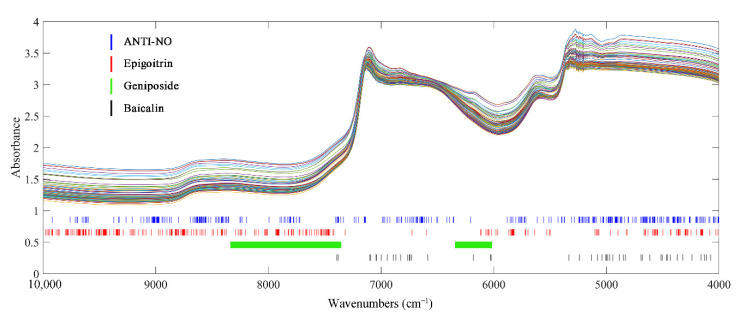
Crucial variables selected of 4 indexes of interest under traditional state.

Synergy interval partial least-squares regression (SIPLS), competitive adaptive reweighted sampling (CARS) and random frog (RF) were applied and compared to select characteristic information correlated with target indexes. For each indicator, the results obtained by different variable screening methods were similar. The optimal screening method was also determined based on the model performance parameters. Models constructed on subsets of variables filtered by different methods are listed in Table S3. The wavenumbers selected by the optimal variable screening method for each indicator are shown in Figure 3.

After variables selection by RF, the spectra of epigoitrin and ANTI-NO retained 250 and 290 variables, respectively. It can be observed that although the number of variables was compressed to less than 20% of the original spectra, the selected key wavenumber points of ANTI-NO were distributed in the full spectra. As explained in the introduction part, it was widely accepted that the biological activity of TCM was the result of the synergistic action of multiple components. Therefore, the key variables corresponding to ANTI-NO indicators were relatively scattered, which represented most of the information of the spectra can be collected to calculate the anti-inflammatory ability. The C=C information contained in the epigoitrin structure was concentrated in the low wavenumber region. The related information of the unique S element and N element in its structure was concentrated in the spectral information in the high wavenumber region of NIR. As for geniposide, the spectra were divided into 18 regions of equal length by SIPLS. A total of 345 variables in the 7th, 11th, 12th, and 13th sub-intervals were selected and combined as a modelling subset. The chosen range around 6100 cm^−1^ corresponded to the absorption bands of C-H in C=C in the iridoid structure of geniposide. The continuous absorption section from 8242 cm^−1^ to 7351cm^−1^ can be attributed to the second overtone region of C-H. The optimal model for baicalin was obtained by using CARS processed spectra, the model of baicalin actually adopted only 47 variables. The variable compression ratio was the highest among the four indexes, which was because baicalin contained a characteristic benzene ring structure. The selected wavenumbers were concentrated in the region from 5400 cm^−1^ to 4000 cm^−1^, which corresponded to the C-H and C-C stretching vibrations in the benzene ring structure. At the same time, the single-strong absorption band at 4065 cm^−1^ caused by C-H stretching and bending vibration was also included. The remaining key variables were scattered around 7000 cm^−1^, which can be attributed to the characteristic absorption of multiple phenolic hydroxyl groups in baicalin.

For the four indicators, the variables screened by the chemometric method were consistent with the characteristics of the indicators. Compared to the number of 1557 variables in the original spectra, the number of variables that need to be considered for subsequent modelling was compressed to less than 25%. Variable screening greatly reduced the amount of computation required for modelling. At the same time, as shown in Table S3, the prediction accuracy of the local models constructed by the chosen variable subsets were higher than that of the global models. It demonstrated that valid information was preserved when redundant variables were removed.

#### 2.3.3. The Results of PLSR Models

**Figure 4:**
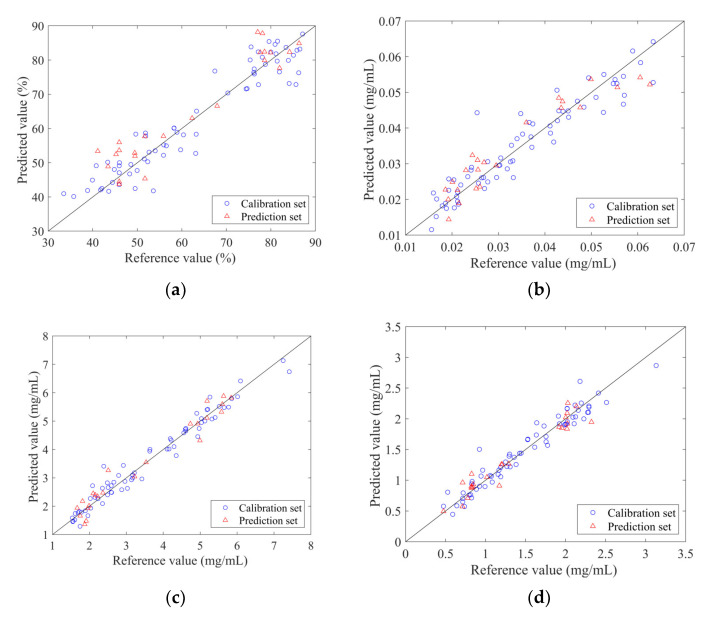
The scatter plot of reference measurements and NIR predictions using the optimal traditional PLSR model: (**a**) ANTI-NO; (**b**) epigoitrin; (**c**) geniposide; (**d**) baicalin.

The PLSR quantitative models were constructed with the selected variable subsets as the input and the reference values measured by traditional methods as the output. The measured and predicted values of the calibration set and prediction set samples for each indicator are displayed in Figure 4. The prediction set samples were scattered and covered within the calibration set samples and the sample points were evenly distributed around y = x.

Besides 36 prediction set samples, the external validation set was applied to verify the accuracy of the PLSR models. In addition to the model parameters, the Wilcoxon rank-sum test was further introduced to test whether there were significant differences between the predicted values and the measured values. The results are displayed in Table 2. *p*-values of four datasets were above 0.05 and the relative standard error of validation (RSEV) values below 20%. It demonstrated that the prediction error value of the model met the application requirements, and there was no significant difference between the two groups of data. For 11 independent samples, the correlation coefficients of validation (Rv) values of ANTI-NO, geniposide and baicalin were all greater than 0.9. However, the Rv value of the PLSR model of epigoitrin was 0.7766, which still needed further consideration before entering the practical application. Predicting target index at low concentration has always been a challenge for NIR applications [19].

### 2.4. Model Construction under Non-Destructive Conditions

#### 2.4.1. Sample Sets Division

Same as the modeling steps for spectra acquired in the traditional state, the non-invasive model construction started with data sets division by SPXY. The results of the division of the dataset are shown in Table 3. It can be observed that the division of the four datasets all met the requirements of model as the distance between the spectra and the reference values were calculated to ensure the rationality of the data division. The external validation set consisted of the original 11 samples. Therefore, the concentration ranges of the validation sets for the three indexes were still outside the calibration sets. It was in line with the situation that may be encountered in the application of the NIR model in Chinese medicine formulations. With the change of production batches, there was a possibility that the concentration range of the new samples will exceed the original dataset.

#### 2.4.2. Spectral Pretreatment and Variables Selection

The model prediction accuracy of PLSR would be affected by the pre-processed methods. Model performances of PLSR after different preprocessing are shown in Table S4. According to the evaluation parameters of the model, when the preprocessing method was MSC, the optimal PLSR models were obtained for ANTI-NO and baicalin. The normalized spectra were optimal for epigoitrin, while the raw spectra without any processing achieved the best model performance for geniposide.

**Figure 5:**
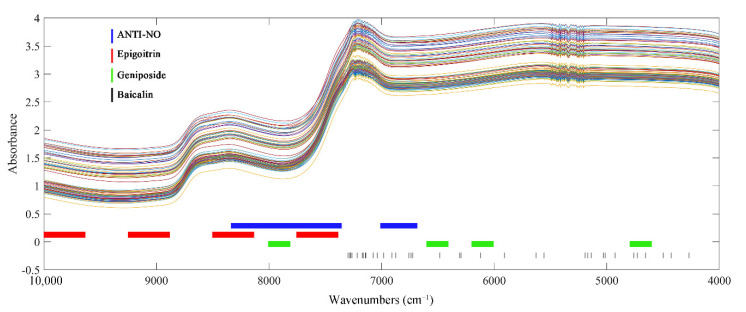
Crucial variables selected of 4 indexes of interest without destroying vial.

Preprocessed spectra of the calibration set were applied as input for variable screening to select key spectral data. The results of variable screening of the four indexes are shown in Figure 5. The optimal variable selection method for ANTI-NO, epigoitrin, and geniposide were all SIPLS with non-invasive spectra as input. As a wavelength interval selection, SIPLS retained the continuity of the spectra by regarding the interval as a unit, and the continuous arrangement and combination of intervals also made it suitable for rapid detection of complex TCM systems. Compared with the traditional model, the effective variables of ANTI-NO retained 55 more variables. However, different from the previous state of being scattered in the whole spectra, the wavenumbers selected by SIPLS were more concentrated. The band region spanning 8000 cm^−1^ corresponds to the second overtone region of C-H. C-H was the structural basis of organic compounds, and information on various indicators of interest can be obtained by analyzing this segment. The selected spectral region below 7000 cm^−1^ was the first overtone region of hydroxyl. This band provided key information for the prediction of anti-inflammatory ability, implying that the biological activity of LOS may be based on hydroxyl-rich substances. The key variables of epigoitrin were concentrated above 7500 cm^−1^. It proved that SIPLS further screened the information scattered in the full spectrum and finally locked the high wavenumber region related to S and N elements to quantify epigoitrin. For geniposide, the key variables dropped from 345 in the traditional model to 208. The added low wavenumber region was derived from the C-C vibration. The spectral region originally spanning 8000 cm^−1^ was compressed to the right of 8000 cm^−1^. However, the spectral region of 6000 cm^−1^ was retained, which proved that C=C in the iridoid structure had an important indication effect the on the construction of the model of geniposide. Without destroying the integrity of the vial, the best subset of variables for baicalin was still selected by CARS, and the number of variables was compressed to 36. It can be clearly observed from the figure that the important wave points of baicalin were consistent with the results in the traditional state. Variables associated with benzene rings and hydroxyl groups were retained for the modelling step. Comparisons of relevant model parameters are listed in Table S5. Compared with the global models, the complexity of the models constructed by selected variables was significantly reduced, and the prediction accuracy was improved. Improvements in model parameters also indicated that the selected key variables were associated with the four indicators.

#### 2.4.3. The Results of PLSR Models

**Figure 6:**
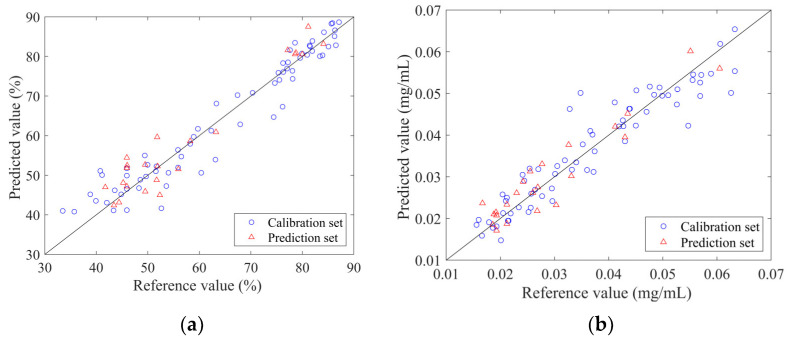

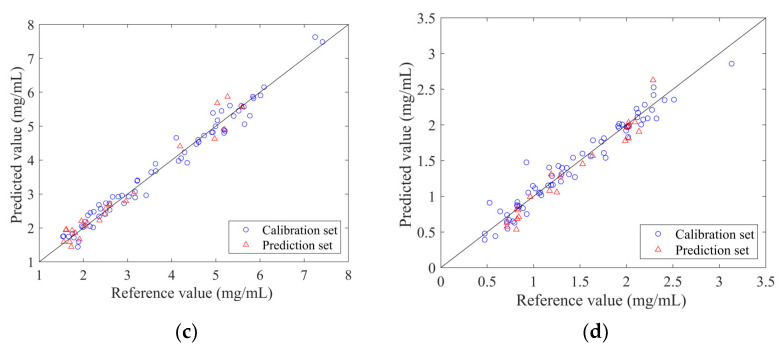
Scatter plot of reference measurements and NIR predictions using the optimal non-invasive PLSR model.: (**a**) ANTI-NO; (**b**) epigoitrin; (**c**) geniposide; (**d**) baicalin.

The PLSR models established by the spectrum penetrating the vial are shown in Figure 6. From the figure, it can be found that the predicted values of the four models were well correlated with the measured values. The sample points were around the line y = x. The validation results of the PLSR model for 11 independent samples are listed in Table 4. The results of the Wilcoxon rank-sum test were satisfactory. It can be observed that the Rv values of the four models were all higher than 0.8, while the RSEV values were less than 20%. These model parameters demonstrated that the established PLSR model can achieve predictions on the validation set samples. The optimal models can be applied to predict new samples.

### 2.5. Comparison of Two System Models

The performance of the PLSR model constructed by the spectra collected by the two acquisition systems is shown in Table 5. Judging from the performance of the samples in the calibration set and prediction set, both models had achieved accurate predictions of the samples. The overall performance was satisfactory and met the application requirements. It can be found from the table that compared with the traditional model, the Rc and Rp values of epigoitrin and baicalin’s non-invasive models were close. The R values of ANTI-NO and geniposide increased slightly. The RSEC and RSEP values of the models constructed by the spectra penetrating the vial, which represented the errors, were all decreased to varying degrees. The residual predictive deviation (RPD) values of the four indicators had increased from 3.2, 3.09, 4.95, and 3.92 in the traditional state to 3.49, 3.10, 5.29, and 4.33, respectively. The improvements of these parameters proved that the prediction error of the model constructed under the non-destructive state was lower than that of the model obtained by the standardized special tubes. The state of the target indexes in the sample system can be more accurately characterized. The results of external validation, listed in Table 2 and Table 4, demonstrated that the prediction accuracy of the non-invasive model for independent samples was as satisfactory as the tedious standardized sweep process that requires breaking the vial and pipetting the sample liquid. Even for epigoitrin with low concentration, the Rv value of the optimal model improved from 0.7766 to 0.8069.

From the model optimization process shown in the supplementary material, it can be found that for both systems, the optimization effect of the model parameters introduced by the spectral preprocessing was far less obvious than that of the variable screening process. Therefore, it was speculated that the advantage of the non-invasive models compared with the models in the standard state mainly came from the wavenumber select process. From the comparison between Figure 3 and Figure 5, it can be observed that the key variables in the non-invasive state had been further compressed or concentrated. As shown in Figure 1C, there were quality fluctuations among different batches of bottles, whereby spectral information irrelevant to the target indexes was introduced into the non-invasive system. Under the perturbation of the disturbance information from the vials, more irrelevant variables were effectively identified and eliminated. Therefore, the input applied by the PLSR model had stronger correlations with the target indicators, which was beneficial to the accuracy of the prediction.

## 3. Materials and Methods

### 3.1. Cell and Reagents

LOS samples of 101 production batches were provided by Yangtze River Pharmaceutical Group (Taizhou, China). The relationship between the production batch number and self-numbering of the sample is shown in Table S1.

Standards of epigoitrin (purity > 99.0%, catalog no: A0529), geniposide (purity > 99.0%, catalog no: A0178) and baicalin (purity > 98.0%, catalog no: A0016) were purchased from Chengdu Must Bio-Technology Co., Ltd. (Chengdu, China). HPLC-grade methanol, acetonitrile and phosphoric acid were obtained from Merck (Darmstadt, Germany). Deionized water was purified by a Milli-Q purification system (Millipore, Bedford, MA, USA).

The murine macrophage RAW 264.7 cell line and ZQ-120 Dulbecco’s modified Eagle medium (DMEM) were purchased from Shanghai Zhong Qiao Xin Zhou Biotechnology Co., Ltd. (Shanghai, China). NO assay kits (S0021) were provided by Beyotime (Nanjing, China). The phosphate-buffered solution was obtained from Labgic Technology Co., Ltd. (Hefei, China). LPS was purchased from Sigma-Aldrich, Inc. (St. Louis, MO, USA).

### 3.2. Sample Preparing

The 35 batches of samples were directly subjected to subsequent spectrum acquisition and reference value acquisition operations without any preparation. The remaining 65 batches of samples were diluted with purified water to extend the range of concerns in the products. The specific dilution schedule is explained in Table S1.

### 3.3. Spectra Acquisition

NIR spectra of prepared samples were collected in a range of 10,000–4000 cm^−1^ by an ANTARIS II (Thermo Scientific, Waltham, MA, USA) in absorbance mode at room temperature. Each spectrum was the average of 32 scans and the average spectrum of 3 times measurements was adopted. All the samples were obtained with air as references and the resolution was set as 8 cm^−1^.

For non-invasive NIR spectra, brown glass vials containing LOS samples were placed directly into the sampling module of the spectrometer. Therefore, the optical path was the diameter of a glass vial, about 12 mm. Spectra collection was also performed in the same state for the empty bottles without liquid. For standardized spectra, the unsealed drug was transferred into a dedicated scanning tube configured with the instrument. The optical path length of the standardized spectra was 4 mm.

### 3.4. Pharmacodynamics Experiment

#### 3.4.1. Cell Culture

The RAW 264.7 cells were cultured with ZQ-120 DMEM in Forma 3111 CO_2_ Incubator (Thermo Scientific, Waltham, MA, USA). Cells were maintained at 37 °C under a 5% CO_2_ atmosphere and relative humidity was controlled at 90%.

#### 3.4.2. Anti-Inflammatory Ability Assay

RAW 264.7 were seeded in 96-well plates (6 × 10^4^ mL^−1^) and cultured at the CO_2_ Incubator. Drug group wells were incubated with different batches of LOS (dilute with medium to 1/100 after spectrum acquisition) and 10 μg mL^−1^ LPS after culturing cells for 24 h. At the same time, 100 μL DMEM medium and 10 μg mL^−1^ LPS were added to the control group and model group wells, respectively. Set up five repetitions per set. The culture medium was collected after 48 h incubation for the NO kits detection.

NO kits worked according to the Griess method. Add equal amounts of Griess Reagent I and Griess Reagent II to the obtained culture medium in turn. Then, the absorbance at 450 nm of the sample obtained by the Spark microplate reader (TECAN) was calculated with a standard calibration curve to confirm the concentration of NO in the sample. The ANTI-NO was calculated by the following formula:

ANTI-NO = (Cm − Cd)/(Cm − Cc) × 100%,(1)

where Cm, Cd, Cc represented the average concentrations of NO in the model group, drug group, and control group samples, respectively.

### 3.5. HPLC Analysis

The samples were diluted 25 times by 40% methanol (*v/v*) and then filtrated through 0.22 µm filter membrane. Standards were dissolved by 40% methanol (*v/v*) to the concentrations of the standard calibration curves in Figure S1.

**Figure 7:**
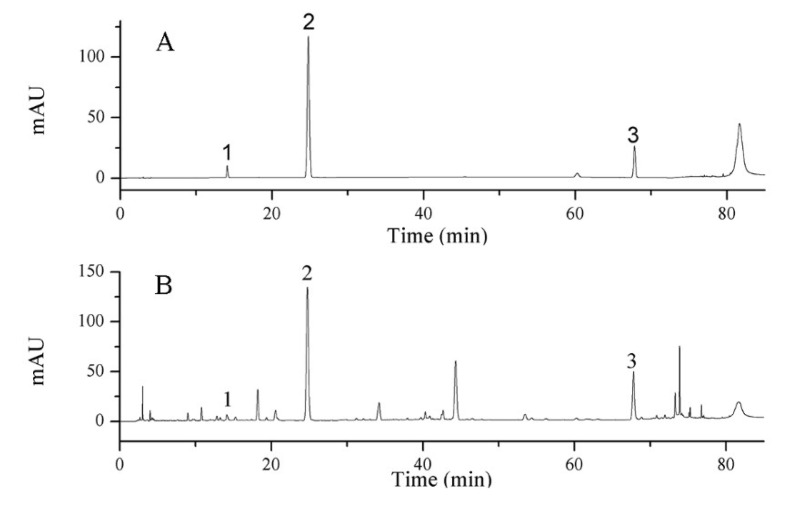
HPLC chromatograms of (**A**) standard solution (1. epigoitrin, 2. geniposide, 3. baicalin) and (**B**) LOS sample.

High-performance liquid chromatograph Agilent 1290 (including quaternary pump, online degassing device, automatic sampler, column temperature controller, DAD detector, and Chem Station) was applied to determine the contents of epigoitrin, geniposide and baicalin. A Luna^®®^ C18 column (250 mm × 4.6 mm, 5 μm) was employed. The mobile phase consisted of acetonitrile (A) and 0.1% phosphoric acid aqueous solution (B) with gradient elution: 0–12 min, 5–11% B; 12–24 min, 11% B; 24–38 min, 11–20% B; 38–62 min, 20% B; 62–70 min, 20–32% B; 70–75 min, 32–80% B; 75–85 min, 80–100% B [20]. The flow rate was set as 1 mL/min, the injection volume was set as 10 μL, and the wavelength was set at 245 nm. Chromatograms are shown in Figure 7.

### 3.6. Chemometrics Methods

#### 3.6.1. Division of Samples

First, 10% of samples (11 batches) were randomly selected to form an external validation set, which was applied to certify the reliability of the established models. The validation set did not participate in the modelling step, it was only used to verify the accuracy of the quantitative models. In order to ensure maximum representation of sample distribution, the remaining 90 samples were divided into the calibration set and the prediction set by the sample set partitioning based on joint x–y distance (SPXY) algorithm in a ratio of 3:1. The distance between samples was calculated simultaneously using x and y variables by the SPXY algorithm to guarantee the representativeness of the calibration set [21].

#### 3.6.2. Spectral Pretreatment

Due to the unavoidable changes in the external environment and sample state, redundant noise information existed in NIR spectra. Spectral pretreatment can help mitigate the effects of non-target factors [22]. In this study, four spectral preprocessing methods, namely normalization, standard normal variate (SNV) transformation, Savitzky–Golay (SG) smoothing and multiplicative scatter correction (MSC) were employed and compared to optimize the model.

#### 3.6.3. Variable Selection Methods

The advantage of information richness of NIR is detrimental to information analysis when the indicators of interest have been specified. An effective variable selection method can eliminate variables irrelevant to the target index while retaining valid information, thereby reducing the difficulty of modeling and improving the accuracy of the model. The following 3 variable selection methods were applied to determine the optimal subset of variables for each index.

SIPLS selected the subintervals of spectra corresponding to the minimum root mean square error of cross-validation (RMSECV) value of local model by dividing and permuting spectral regions [23]. SIPLS followed the principle that NIR spectra had continuous features of bands, while wavenumber point selection efficiently screened key variables based on mathematical principles [24]. CARS simulated the principle of survival of the fittest in Darwin’s evolution theory. In this process, Monte Carlo sampling was used to construct the local model. The wavenumbers with small absolute values of regression coefficients in the model were continuously eliminated, and finally, the optimal subset of variables was selected according to the RMSECV value [25]. RF calculates the importance of each variable by the probability that each variable is selected in the model space [26].

#### 3.6.4. PLSR

PLSR is the most extensive quantitative regression method of NIRs rapid detection system [27]. The performance of the model was affected by the setting of the number of LVs. In this study, LVs was determined according to RMSECV by leave-one-out cross-validation.

#### 3.6.5. Evaluation Criteria of Models

Performance of constructed PLSR model was evaluated by 8 indexes, namely: Rc, Rp, RSEC, RSEP, RMSEC, RMSEP, RPD and RMSECV. The results of the external test are mainly judged by correlation coefficients of Rv, RSEV, and RMSEV. At the same time, the Wilcoxon rank-sum test was introduced to further compare the reference value and the model prediction value of the validation set samples to prove the prediction accuracy of the obtained model.

In general, the value of correlation coefficients should be close to 1. The relative standard error values of the 3 sample sets were expected to be small and close to each other. Moreover, the RPD value greater than 3 was the requirement of the optimal model [28].

### 3.7. Software

For NIRs data acquisition, TQ Analyst 8.0 were applied. The data processing and graphic drawing were performed by MATLAB software (version 2018b, Mathworks, Natick, MA, USA).

## 4. Conclusions

In this study, high-performance and non-invasive quantitative models of anti-inflammatory bioactivity and three key chemical indicators including epigoitrin, geniposide, and baicalin were developed based on NIRs and chemometrics methods. The Rc values of the non-destructive system were greater than 0.94 and the RSEC values were lower than 15% for four indexes. Compared with the standard system, the constructed model through vials achieved higher prediction accuracy. The obtained models can be applied to the quality detection of LOS products instead of traditional analytical methods to improve production efficiency. This study was the first to verify the anti-inflammatory ability of LOS at the cellular level and realized its non-destructive and rapid detection. Due to the complexity of the efficacy of TCM, it was difficult to comprehensively characterize the quality of drugs by purely chemical indicators. Therefore, the introduction of anti-inflammatory indicators as testing objects can improve the understanding of the quality of TCM formulations. The multi-indicator advantage of the NIRs can realize the comprehensive detection of TCM, and its fast and non-destructive characteristics provide feasibility for high-throughput analysis. The NIR non-destructive detection system was conducive to controlling the quality of LOS formulations, ensuring the effectiveness and safety of drugs, and can provide a reference for the quality control of other TCM formulations.

## Figures and Tables

**Table 1:** Reference values for 4 indexes in the data sets.

Data Sets.		Sample Number	Minimum Concentration (% or mg/mL)	Maximum Concentration (% or mg/mL)	Mean (% or mg/mL)	Std
ANTI-NO	Calibration set	67	33.49	87.12	62.87	0.1626
	Prediction set	23	41.13	86.33	61.93	0.1620
	Validation set	11	44.26	88.97	69.77	0.1644
Epigoitrin	Calibration set	67	0.0156	0.0633	0.0354	0.0138
	Prediction set	23	0.0186	0.0626	0.0334	0.0142
	Validation set	11	0.0185	0.0702	0.0471	0.0151
Geniposide	Calibration set	67	1.537	7.413	3.609	1.539
	Prediction set	23	1.668	5.840	3.424	1.609
	Validation set	11	1.421	7.032	4.602	1.842
Baicalin	Calibration set	67	0.4739	3.131	1.493	0.6037
	Prediction set	23	0.4742	2.323	1.354	0.6001
	Validation set	11	0.5729	2.820	1.746	0.7158

**Table 2:** The results of external validation of traditional models.

Sample No.	ANTI-NO		Epigoitrin		Geniposide		Baicalin	
	Reference Value	Predicted Value	Reference Value	Predicted Value	Reference Value	Predicted Value	Reference Value	Predicted Value
1	0.4426	0.4917	0.0425	0.02952	2.396	2.632	0.7825	0.8637
2	0.8427	0.8541	0.0702	0.07235	5.63	5.692	2.421	2.171
3	0.6185	0.5988	0.0381	0.02531	4.221	2.897	1.303	1.421
4	0.5161	0.5743	0.0305	0.02882	2.631	2.094	1.295	1.172
5	0.6956	0.6698	0.0452	0.03126	4.054	3.738	1.706	1.451
6	0.4594	0.4362	0.0185	0.01739	1.421	1.239	0.5729	0.6174
7	0.7785	0.8132	0.0468	0.05402	5.027	5.152	1.608	1.712
8	0.8735	0.8326	0.0616	0.06644	6.525	6.937	2.82	2.366
9	0.7952	0.9022	0.0466	0.04715	6.437	6.279	2.413	2.449
10	0.8897	0.7868	0.0648	0.04770	5.248	4.650	2.014	2.020
11	0.7633	0.7989	0.053	0.05547	7.032	6.399	2.269	2.652
Rv	0.9356		0.7766		0.9516		0.9468	
RMSEV ^1^	0.055		0.009		0.540		0.220	
RSEV	7.7%		18.5%		11.0%		11.7%	
*p*	0.8955		0.7928		0.7427		1	

^1^ RMSEV: the root mean square error of validation.

**Table 3:** Reference values for 4 indexes in the data sets.

Data Sets		Sample Number	Minimum Concentration (% or mg/mL)	Maximum Concentration (% or mg/mL)	Mean (% or mg/mL)	Std
ANTI-NO	Calibration set	67	33.49	87.12	64.38	0.1642
	Prediction set	23	41.76	84.10	57.51	0.1453
	Validation set	11	44.26	88.97	69.77	0.1644
Epigoitrin	Calibration set	67	0.0156	0.0633	0.0368	0.0140
	Prediction set	23	0.0167	0.0605	0.0293	0.0119
	Validation set	11	0.0185	0.0702	0.0471	0.0151
Geniposide	Calibration set	67	1.537	7.413	3.799	1.537
	Prediction set	23	1.558	5.600	2.871	1.401
	Validation set	11	1.421	7.032	4.602	1.842
Baicalin	Calibration set	67	0.4739	3.131	1.462	0.6193
	Prediction set	23	0.7137	2.286	1.443	0.5637
	Validation set	11	0.5729	2.820	1.746	0.7158

**Table 4:** The results of external validation of traditional models.

Sample No.	ANTI-NO		Epigoitrin		Geniposide		Baicalin	
	Reference Value	Predicted Value	Reference Value	Predicted Value	Reference Value	Predicted Value	Reference Value	Predicted Value
1	0.4426	0.4728	0.0425	0.02919	2.396	2.056	0.7825	0.979
2	0.8427	0.9373	0.0702	0.07578	5.63	6.118	2.421	2.518
3	0.6185	0.6515	0.0381	0.03811	4.221	3.496	1.303	1.700
4	0.5161	0.5683	0.0305	0.03752	2.631	2.755	1.295	1.557
5	0.6956	0.6996	0.0452	0.04223	4.054	3.847	1.706	1.796
6	0.4594	0.4520	0.0185	0.01870	1.421	1.466	0.5729	0.629
7	0.7785	0.7173	0.0468	0.03451	5.027	5.126	1.608	1.585
8	0.8735	0.9306	0.0616	0.07149	6.525	6.323	2.82	2.623
9	0.7952	0.8521	0.0466	0.05203	6.437	5.420	2.413	2.427
10	0.8897	0.8075	0.0648	0.04884	5.248	4.977	2.014	2.099
11	0.7633	0.8206	0.053	0.05317	7.032	5.813	2.269	2.286
Rv	0.9349		0.8069		0.9457		0.9670	
RMSEV	0.056		0.008		0.571		0.174	
RSEV	7.8%		17.3%		11.6%		9.3%	
*p*	0.6936		0.7928		0.6458		0.6936	

**Table 5:** The optimal PLSR model of 4 indicators.

Analytes	M. T. ^1^	P. M. ^2^	V. S. M. ^3^	V. N. ^4^	LVs ^5^	Rc	RMSEC ^6^	RSEC	Rp ^7^	RMSEP ^8^	RSEP	RPD
ANTI-NO	T ^9^	Normalization	RF	290	9	0.9526	0.049	7.6%	0.9296	0.058	9.1%	3.20
	N ^10^	MSC	SIPLS	345	11	0.9658	0.042	6.4%	0.9524	0.043	7.3%	3.49
Epigoitrin	T ^9^	SG smoothing	RF	250	8	0.9434	0.005	12.0%	0.9439	0.005	12.7%	3.09
	N ^10^	Normalization	SIPLS	388	11	0.9409	0.005	12.0%	0.9437	0.004	12.2%	3.10
Geniposide	T ^9^	MSC	SIPLS	345	13	0.9820	0.289	7.4%	0.9791	0.320	8.5%	4.95
	N ^10^	Raw	SIPLS	208	8	0.9885	0.231	5.6%	0.9814	0.263	8.3%	5.29
Baicalin	T ^9^	Normalization	CARS	47	10	0.9680	0.150	9.4%	0.9669	0.150	10.1%	3.92
	N ^10^	MSC	CARS	36	7	0.9735	0.141	8.9%	0.9652	0.144	9.3%	4.3

^1^ M.T.: model type. ^2^ P.M.: pretreatment methods. ^3^ V. S. M.: variables selection methods. ^4^ V. N.: variable numbers. ^5^ LVs: latent variables. ^6^ RMSEC: root mean square error of calibration. ^7^ Rp: correlation coefficients of prediction. ^8^ RMSEP: the root mean square error of prediction. ^9^ T.: traditional model. ^10^ N.: non-invasive model.

## Data Availability

Not applicable.

## Supplementary Materials

The following supporting information can be downloaded at: https://www.mdpi.com/article/10.3390/molecules27092955/s1, Table S1: Sample production batches and dilution arrangements; Table S2: Performance of different spectral pretreatments models for 4 indexes under standard conditions; Table S3 Performance of different variable selection models for 4 indexes under standard conditions; Table S4: Performance of different spectral pretreatments models for 4 indexes under non-destructive conditions.; Table S5: Performance of different variable selection models for 4 indexes under non-destructive conditions; Figure S1: Concentrations, and standard calibration curves.

## Sample Availability

Samples of the compounds are available from the authors.
