# Supplementary material for: Non-Invasive Detection of Anti-Inflammatory Bioactivity and Key Chemical Indicators of the Commercial Lanqin Oral Solution by Near Infrared Spectroscopy
Source: Molecules. 2022 May 5;27(9):2955. doi: 10.3390/molecules27092955 (PMC9099839; doi:10.3390/molecules27092955)
Supplement: Supplementary file 1 [file molecules-27-02955-s001.zip › molecules-1692597-supplementary.pdf]

Supplementary Materials

Non-Invasive Detection of Anti-Inflammatory Bioactivity and Key Chemical Indicators of the Commercial Lanqin Oral Solution by Near Infrared Spectroscopy

Hui Ma, Lulu Xiao, Dongchen Xu, Yingrui Geng, Xuesong Liu, Yong Chen * and Yongjiang Wu *

College of Pharmaceutical Sciences, Zhejiang University, Hangzhou 310058, China; 12119013@zju.edu.cn (H.M.); 22119162@zju.edu.cn (L.X.); xdcgg@zju.edu.cn (D.X.); 22019084@zju.edu.cn (Y.G.); liuxuesong@zju.edu.cn (X.L.)

* Correspondence: chenyong1@zju.edu.cn (Y.C.); yjwu@zju.edu.cn (Y.W.)

Table S1. Sample production batches and dilution arrangements.

Sample number	Production batch	Dilution factor *	Sample number	Production batch	Dilution factor *
1	19070182	1	52	21022081	1
2	19070281	2.08	53	21021482	2.62
3	19070282	1.03	54	21022281	1.57
4	19070481	1	55	21022282	1
5	19070581	2.05	56	21022481	1.42
6	19070681	1	57	21022582	1
7	19070881	2.14	58	21022581	2.53
8	19070882	1.09	59	21022681	1.63
9	19070982	1	60	21022782	1
10	19071181	1.96	61	21022781	2.71
11	19071281	1	62	21030181	1.36
12	19071282	2.2	63	21030281	1
13	19071381	1.15	64	21030282	2.47
14	19071582	1	65	21030381	1.69
15	19071681	2.98	66	21030481	1
16	19071682	1.9	67	21030482	2.77
17	19071782	1	68	21030581	1.3
18	19071881	2.26	69	21030681	1
19	19071882	1.21	70	21030682	2.41
20	19072081	1	71	21030881	1.75
21	19072082	2.92	72	21031081	1
22	19072282	1.84	73	21031082	2.83
23	19072382	1	74	21031181	1.24
24	19072581	2.32	75	21031281	1
25	19072582	1.27	76	21031282	2.35
26	19072682	1	77	21031381	1.81
27	19072781	2.86	78	21031581	1
28	19072782	1.78	79	21031582	2.89
29	19072982	1	80	21031781	1.48
30	19073081	2.38	81	21031881	1
31	19073181	1.33	82	21031882	2.29
32	19080281	1	83	21070681	1.87
33	19080282	2.8	84	21070781	1

34	19080381	1.72	85	21070782	2.95
35	19080481	1	86	21070881	1.18
36	19080581	2.44	87	21070981	1
37	19080682	1.39	88	21070982	2.23
38	19080782	1	89	21071081	1.93
39	19080881	2.74	90	20123131	1.06
40	19080982	1.66	91	21072981	1
41	19081081	1	92	21073081	3.01
42	19081282	1.45	93	21080181	1.12
43	21021481	1	94	21081082	1
44	21021582	2.68	95	21081181	2.17
45	21021581	1.6	96	21080881	1.99
46	21021682	1	97	21080982	1
47	21021781	2.56	98	21081282	3.07
48	21021782	1.51	99	21081381	2.02
49	21021882	1	100	21081781	1
50	21021981	2.59	101	21081782	2.11
51	21021982	1.54			

* 1 means no dilution is done.

Table S2. Performance of different spectral pretreatments models for 4 indexes under standard conditions.

Analytes	Pretreatment	LVs	Rc	RMSEC	RSEC	Rp	RMSEP	RSEP	RPD
ANTI-NO	Raw	6	0.9305	0.059	9.1%	0.9039	0.068	10.6%	2.68
	**Normalization**	**10**	**0.9491**	**0.051**	**7.8%**	**0.9181**	**0.063**	**9.8%**	**2.90**
	SNV	5	0.9315	0.059	9.0%	0.9062	0.067	10.5%	2.79
	SG smoothing	6	0.9302	0.059	9.1%	0.9060	0.067	10.5%	2.71
	MSC	5	0.9316	0.059	9.0%	0.9059	0.067	10.5%	2.79
Epigoitrin	Raw	10	0.8989	0.006	15.8%	0.9193	0.005	15.1%	2.55
	Normalization	10	0.9056	0.006	15.3%	0.9142	0.006	15.6%	2.47
	SNV	10	0.9100	0.006	15.0%	0.9103	0.006	15.9%	2.42
	**SG smoothing**	**8**	0.9119	**0.006**	**14.8%**	**0.9218**	**0.005**	**14.9%**	**2.58**
	MSC	10	0.9084	0.006	15.1%	0.9245	0.005	14.6%	2.63
Geniposide	Raw	17	0.9802	0.303	7.7%	0.9774	0.333	8.8%	4.73
	Normalization	16	0.9799	0.304	7.8%	0.9739	0.357	9.5%	4.44
	SNV	13	0.9737	0.348	8.9%	0.9728	0.364	9.7%	4.35
	SG smoothing	10	0.9737	0.348	8.9%	0.9650	0.412	10.9%	3.83
	**MSC**	**15**	0.9803	**0.302**	**7.7%**	**0.9787**	**0.323**	**8.6%**	**4.87**
Baicalin	Raw	4	0.9265	0.209	13.6%	0.8813	0.335	19.9%	2.16
	**Normalization**	**3**	**0.9203**	**0.217**	**14.1%**	**0.8986**	**0.311**	**18.5%**	**2.33**
	SNV	5	0.9351	0.196	12.8%	0.8741	0.345	20.5%	2.12
	SG smoothing	5	0.9284	0.206	13.4%	0.8561	0.367	21.8%	1.97
	MSC	5	0.9350	0.197	12.8%	0.8739	0.345	20.5%	2.12

Table S3. Performance of different variable selection models for 4 indexes under standard conditions.

Analytes	V. S. M.[1]	V. N.[2]	LVs	Rc	RMSEC	RSEC	Rp	RMSEP	RSEP	RPD
ANTI-NO	CARS	71	10	0.9594	0.046	7.0%	0.9046	0.068	10.6%	2.52
	SIPLS	311	7	0.9361	0.057	8.7%	0.8896	0.072	11.3%	2.40
	**RF**	**290**	**9**	**0.9526**	**0.049**	**7.6%**	**0.9296**	**0.058**	**9.1%**	**3.20**
Epigoitrin	CARS	62	7	0.9328	0.005	13.0%	0.9213	0.005	14.9%	2.62
	SIPLS	310	5	0.8901	0.006	16.4%	0.9298	0.005	14.1%	2.72
	**RF**	**250**	**8**	**0.9434**	**0.005**	**12.0%**	**0.9439**	**0.005**	**12.7%**	**3.09**
Geniposide	CARS	34	10	0.9745	0.342	8.7%	0.9573	0.455	12.1%	3.47
	**SIPLS**	**345**	**13**	0.9820	**0.289**	**7.4%**	**0.9791**	**0.320**	**8.5%**	**4.95**
	RF	250	10	0.9648	0.402	10.2%	0.9748	0.351	9.3%	4.49
Baicalin	**CARS**	**47**	**10**	**0.9680**	**0.150**	**9.4%**	**0.9669**	**0.150**	**10.1%**	**3.92**
	SIPLS	272	10	0.9409	0.203	12.6%	0.9636	0.157	10.6%	4.44
	RF	250	10	0.9395	0.205	12.8%	0.9715	0.139	9.4%	4.79

[1] V. S. M.: Variables Selection Method; [2] V. N.: Variable numbers.

Table S4. Performance of different spectral pretreatments models for 4 indexes under non-destructive conditions.

Analytes	Pretreatment.	LVs	Rc	RMSEC	RSEC	Rp	RMSEP	RSEP	RPD
ANTI-NO	Raw	10	0.9538	0.049	7.4%	0.9509	0.053	7.4%	2.69
	Normalization	10	0.9557	0.048	7.2%	0.9499	0.054	7.5%	2.64
	SNV	9	0.9554	0.048	7.2%	0.9529	0.049	7.3%	2.92
	SG20	11	0.9463	0.053	7.9%	0.9512	0.049	7.4%	2.93
	**MSC**	**9**	**0.9558**	**0.048**	**7.2%**	**0.9529**	**0.050**	**7.3%**	**2.91**
Epigoitrin	Raw	12	0.9614	0.004	9.7%	0.9285	0.004	13.7%	2.70
	**Normalization**	**13**	**0.9619**	**0.004**	**9.7%**	**0.9221**	**0.005**	**14.3%**	**2.67**
	SNV	10	0.9465	0.004	11.4%	0.9213	0.005	14.4%	2.57
	SG20	14	0.9296	0.005	13.0%	0.9009	0.005	16.0%	2.30
	MSC	10	0.9526	0.004	10.8%	0.9071	0.005	15.5%	2.38
Geniposide	**Raw**	**10**	**0.9711**	**0.364**	**8.9%**	**0.9585**	**0.370**	**12.3%**	**3.72**
	Normalization	10	0.9672	0.387	9.5%	0.9624	0.429	11.7%	3.23
	SNV	8	0.9674	0.386	9.4%	0.9663	0.428	11.1%	3.23
	SG8	11	0.9651	0.399	9.8%	0.9642	0.512	11.4%	2.75
	MSC	8	0.9685	0.380	9.3%	0.9662	0.407	11.1%	3.40
Baicalin	Raw	11	0.9686	0.153	9.6%	0.9495	0.173	11.2%	3.55
	Normalization	11	0.9550	0.182	11.5%	0.9623	0.150	9.7%	3.97
	SNV	9	0.9710	0.147	9.3%	0.9622	0.150	9.7%	4.11
	SG5	11	0.9517	0.189	11.9%	0.9553	0.163	10.6%	3.71
	**MSC**	**10**	**0.9759**	**0.134**	**8.5%**	**0.9570**	**0.160**	**10.4%**	**3.82**

Table S5. Performance of different variable selection models for 4 indexes under non-destructive conditions.

Analytes	V. S. M.[1]	V. N.[2]	LVs	Rc	RMSEC	RSEC	Rp	RMSEP	RSEP	RPD
	CARS	50	6	0.9440	0.054	8.1%	0.9404	0.048	8.2%	2.99
ANTI-NO	**SIPLS**	**345**	**11**	**0.9658**	**0.042**	**6.4%**	**0.9524**	**0.043**	**7.3%**	**3.49**
	RF	50	8	0.9671	0.041	6.2%	0.9290	0.053	8.9%	2.78
	CARS	47	10	0.9851	0.002	6.1%	0.9514	0.004	11.4%	3.25
Epigoitrin	**SIPLS**	**388**	**11**	**0.9409**	**0.005**	**12.0%**	**0.9437**	**0.004**	**12.2%**	**3.10**
	RF	110	5	0.9748	0.003	7.9%	0.8911	0.004	16.8%	3.16
	CARS	39	8	0.9705	0.367	9.0%	0.9658	0.337	11.2%	4.07
Geniposide	**SIPLS**	**208**	**8**	**0.9885**	**0.231**	**5.6%**	**0.9814**	**0.263**	**8.3%**	**5.29**
	RF	80	6	0.9790	0.311	7.6%	0.9674	0.340	10.9%	4.06
	**CARS**	**36**	**7**	**0.9735**	**0.141**	**8.9%**	**0.9652**	**0.144**	**9.3%**	**4.33**
Baicalin	SIPLS	215	6	0.9485	0.195	12.3%	0.9391	0.189	12.3%	3.24
	RF	140	6	0.9616	0.169	10.6%	0.9433	0.183	11.9%	3.34

[1] V. S. M.: Variables Selection Method; [2] V. N.: Variable numbers.

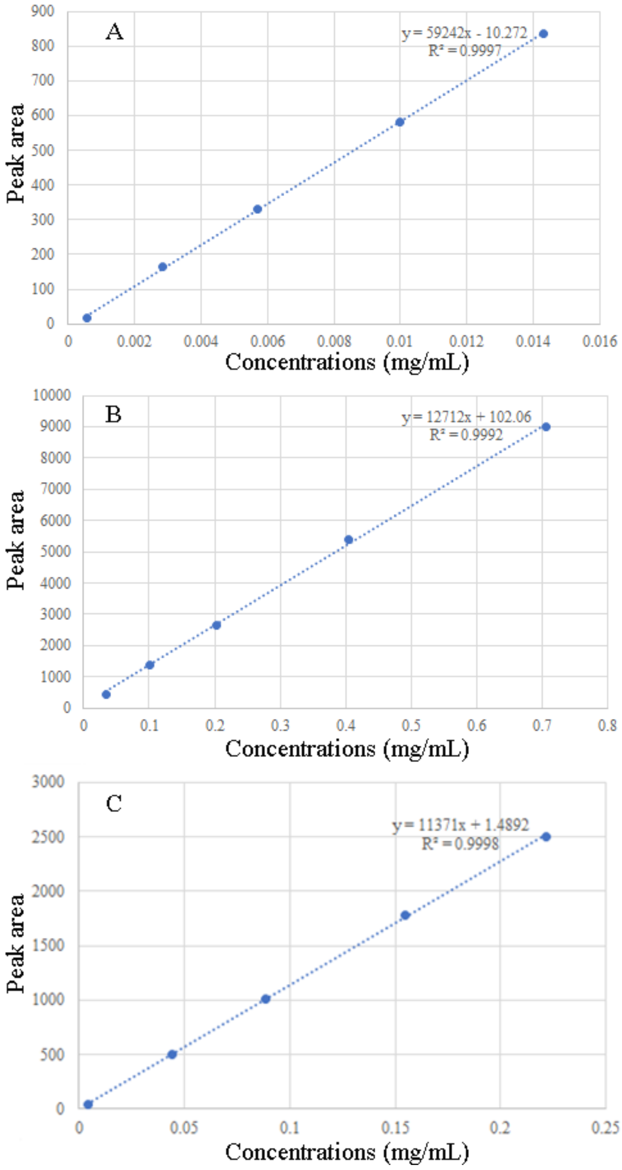

Figure S1. Concentrations, and standard calibration curves (**A**) Epigoitrin, (**B**) Geniposide, (**C**) Baicalin.
